# Polyurethane: An Old Material for a New Generation of Antibiotic Spacer Implants

**DOI:** 10.1016/j.artd.2024.101409

**Published:** 2024-08-09

**Authors:** James W. Pritchett

**Affiliations:** Swedish Orthopedics, Seattle, WA, USA

**Keywords:** Polyurethane, Prosthetic joint infection, Antibiotics, Hip, Knee

## Abstract

**Background:**

Polyurethane tibial and acetabular inserts that release high concentrations of antibiotics were used with debridement and implant retention to treat prosthetic joint infections. The hypothesis was that a low-friction, antibiotic-releasing bearing could provide a simpler, safer, and more patient-accepted treatment for infection using antibiotic cement and intravenous antibiotics.

**Methods:**

Patients (n = 106) with culture-positive infections received antibiotic inserts. Vancomycin and tobramycin were mixed into the polyurethane polymer at 7% by weight. Contraindications to debridement antibiotics and implant retention were a sinus tract, loose prostheses, and/or the wound could not be closed. Measurable outcomes were success in controlling infection, complications, patient acceptable symptomatic state, and need for revision surgery. Antibiotic levels were measured in joint fluid and blood; laboratory mechanical wear tests were performed; and results were compared to bone cement and polyethylene containing antibiotics.

**Results:**

Antibiotic-infused spacers sustained joint fluid antibiotic levels 8-12 times the therapeutic level and produced low serum levels with no toxicities. Mechanical testing showed low wear and retained mechanical integrity. All patients achieved complication-free remission of infection at a follow-up of 5-26 years. All patients had Harris hip and Knee Society scores above 85, and 68% achieved patient acceptable symptomatic state.

**Conclusions:**

All patients achieved remission of infection, fewer complications compared to revision using antibiotic bone cement, no antibiotic toxicity or adverse drug reactions, and 68% achieved patient acceptance. The antibiotic polyurethane inserts provided antibacterial efficacy comparable with currently used bone cement spacers, and their wear rate was approximately 20 times lower than bone cement as an articulation.

## Introduction

Currently, the method of treatment for prosthetic joint infection (PJI) is driven by the resources available, the experience of the surgical team, and the preferences of the patient and surgeon. Treating a PJI with a one-stage procedure that retains rather than removes a well-placed implant is an attractive alternative to a 1-stage or 2-stage revision. The goal of this study was to evaluate a simpler, less traumatic, and more patient-centric alternative to 1-stage or 2-stage explant and reimplantation surgery. Debridement antibiotics and implant retention (DAIR), although popular with patients and surgeons, has not been entirely successful [1,2]. A drug-eluting bearing surface might increase the efficacy and provide a more successful option for treating PJI [3,4].

Patients have described 1-stage and 2-stage reimplantation procedures as profoundly negative experiences with major physical, social, and emotional downsides [5]. Surgeons describe the experience of PJI as emotionally difficult, as in their efforts to help their patients, harm has occurred instead [6]. The current PJI treatment methods are burdensome. Chronic suppression with antibiotics can be an effective strategy for some patients; however, some do not feel well with systemic antibiotics either for the short term or long term because of their effects on the microbiome. A method that avoids systemic antibiotics is an attractive option.

Using bone cement containing antibiotics is effective in producing high antibiotic concentrations in the tissues. Bone cement has been used since 1953 as an adhesive and bearing surface [7], although it is too abrasive to be useful long-term as a bearing surface. Bone cement breaks down over time and can produce squeaking and wear debris when used as a bearing surface. Also, bone cement spacers have poor wear resistance and can fracture in up to 12% of cases in the short term and in 60% of cases if left on a permanent basis [3,8,9]. Because of these issues, many surgeons opt to use antibiotic cement with cobalt-chromium femoral components and all polyethylene (PE) for the acetabular or tibial bearing that is cemented directly to the bone using antibiotic cement as their spacer choice [3].

Clinical infection results when the balance tips toward the bacterial burden. When the balance tips in favor of the immune system, the infection goes into remission [10]. Sustaining a consistently high antibiotic level in contact with the infected joint implant for several weeks has been the challenge in achieving and maintaining remission of PJI. The antibiotic levels necessary to penetrate biofilm are higher than those needed to penetrate tissue [2,11]. This has been a reason DAIR procedures have not been effective consistently. Antibiotic cements deliver very high antibiotic levels, but only for a short time [12,13]. Intra-articular infusion can produce extremely high antibiotic levels, but this is a cumbersome and demanding regimen requiring an indwelling catheter that must be maintained daily and then removed [11]. An antibiotic-releasing bearing surface that does not require ongoing care is an attractive concept that could make DAIR procedures more effective [3,4].

Polyurethane (PUR) has been a candidate material for implant arthroplasty both as a bearing surface and as a bone cement [[14], [15], [16]]. It was initially used as a “bone glue” for fractures and occasionally for fusions [17]. Despite its early success, it devolved into complications and was discredited and abandoned [18]. The initial PUR preparations were crude. Recent preparations have been more successful in load-bearing applications [2,12,15]. It is possible to embed antibiotics in PUR and have elution into the tissues with time and use. The goal is to create a bearing surface that is safe and effective mechanically for the long term and that also releases antibiotics in high enough levels to obtain remission of PJI. Therefore, antibiotic polyurethane (aPUR) would be useful as both a joint arthroplasty bearing surface and as an effective treatment for infection.

This study asked: (1) Is aPUR a safe and effective material for use as a bearing surface in joint arthroplasty? (2) Can aPUR reliably induce and maintain a remission for PJI without the need for additional revision surgery or supplemental treatment? (3) Is aPUR a better alternative to a cement spacer or an antibiotic-cemented all PE tibial and acetabular prostheses?

## Material and methods

**Figure 1:**
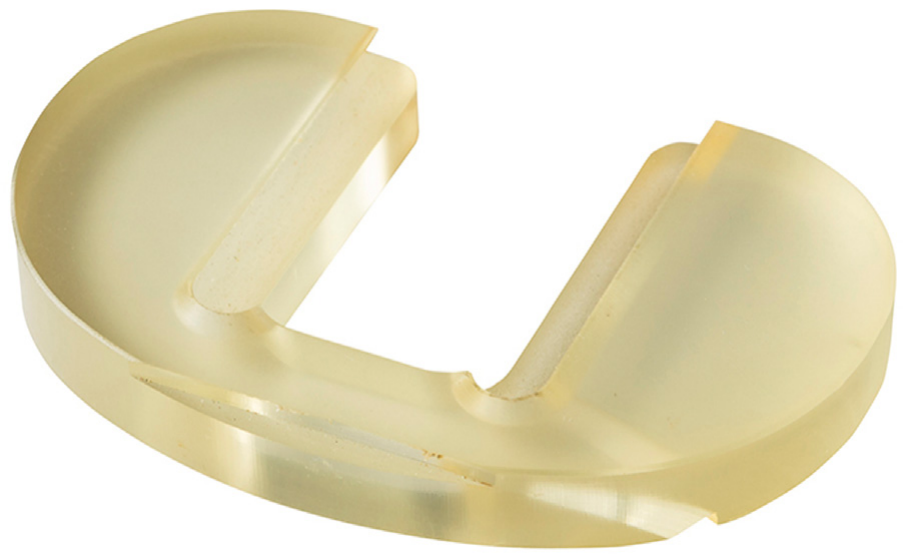
Photograph of an antibiotic tibial polyurethane insert.

**Figure 2:**
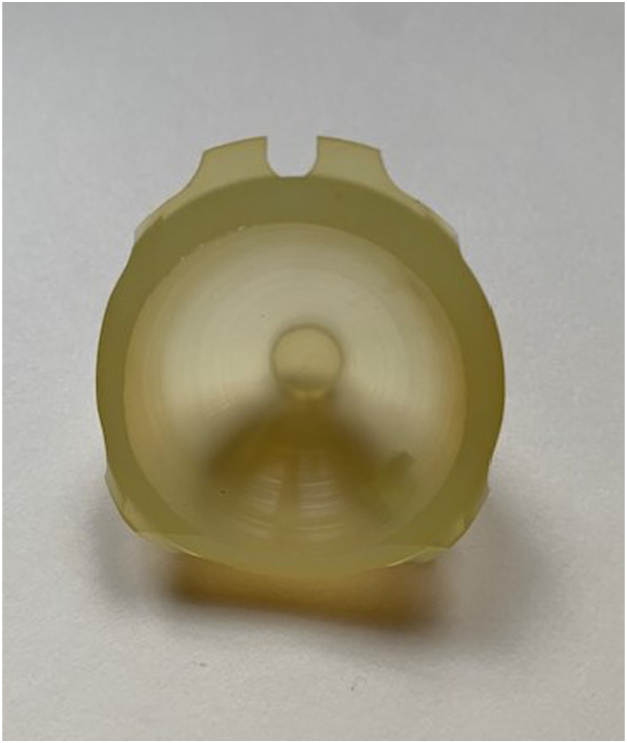
Photograph of an antibiotic acetabular polyurethane insert.

The institutional review board approved this study. The PUR elastomer tested has been described in previous studies [19,20]. This polycarbonate PUR was selected based on load-bearing capacity and resistance to wear. The PURs were machined into tibial inserts (Fig. 1) and acetabular liners (Fig. 2). The inner and outer dimensions were machined to match the acetabular shell or metal tibial backing of the specific joint needed based on the specific molds from the implant manufacturer (Signal Medical, Marysville, MI). The aPUR inserts are done under the compassionate use provisions rather than cleared for use. The femoral counterface of either the hip or knee was ceramic, titanium nitride-coated titanium, or cobalt chromium. The inserts were made using phase compression molding. Testing was performed according to ASTM F732-17 [21].

Tobramycin and vancomycin were provided in powder form and embedded in the PUR during preparation using the prepolymer method as described previously [16,19]. Calcium sulfate beads containing antibiotics are an option but were not used in this study. The antibiotic PE samples for comparison were embedded with antibiotics using previously described techniques [3]. The calculation for the release exponent indicated that 7%-9% by weight was the safe and effective concentration. Testing was performed using the AMTI KS-2-6-1000 simulator for knees and the MTS for hips (MTS System, Eden Prairie, MN) [[10], [11], [22], [23]]. Wear simulator testing was performed according to ISO 14243-1:200. One knee and one hip insert served as a soak control, and 5 tibial and 5 acetabular inserts were tested fully. Lubrication was with diluted calf serum, and the wear test was performed at 37°C ± 1°C. PUR is hydrophilic compared to PE. PUR wear tests have been done using calf serum as the lubricant. The tested implants were removed from their original sterile packing and treated with accelerated oxidative aging according to ASTM F2003 [24]. Their initial dry weight was recorded. Prior to the wear simulation testing, the tibial and acetabular inserts were soaked in serum and measured weekly until there was less than a 10% weight change. They were soaked for a minimum of 2 weeks until there was no further weight change. The longest soaking interval was 4 weeks.

Next, the implants were presoaked for 7 days in the lubricant, cleaned, and weighed again. The simulation was continued for a total of 10 × 10^6^ cycles at a frequency of 1 Hz. For every 0.5 × 10^6^, the components were measured gravimetrically according to ISO 14243-1:200 and ASTM F2025 [7,20,25]. Two methods were used. In method 1, the average wear rate was determined by a linear regression of the implants’ weight over time. In method 2, the total weight was calculated by subtracting the final weight after complete drying from its initial weight and dividing by 10 million cycles (Mc). The volumetric wear can be determined using the density of PUR (1.19 g/mL).

Mechanical (tensile) tests were performed according to ASTM D638. Standard testing using a universal testing machine (UTM-66A, Japan) was used. Tests were performed at 50 mm/min with a load cell of 100 N using dumbbell-shaped samples; 5 samples were tested.

### Patients

There were 106 patients in the study who received the antibiotic inserts (54 men, 50 women, and 2 nonbinary) with a mean age of 61 years (range, 44-76). These patients were treated using the DAIR technique. After a thorough debridement, pulse lavage was used with 5 liters of saline, and 1 liter of dilute chlorhexidine was left in the wound for 3 minutes, followed by irrigation with another liter of saline. The surgeons changed gowns and gloves and used a separate set of instruments to place the aPUR and close the incision.

All patients provided their informed consent for participation in their treatment of this study and for publication of the results. All patients had a culture-positive infection with laboratory tests confirming infection (Table 1). The Musculoskeletal Infection Society criteria were used to determine infection. No patients had infections that were resistant to vancomycin and tobramycin. All patients were symptomatic, and their infections were identified from 19 days to 186 days following surgery. Both acute and chronic infections were treated. All patients were followed at least annually, and none were lost to follow-up. The mean follow-up was 10 years (range, 5-26 years).

**Table 1:** Microorganism frequency.

Microorganism	Number (%)
Aerobic gram-positive bacteria	82 (77)
Coagulase-negative Staphylococcus species (other than *S. lugdunensis*)	37 (35)
S. aureus	24 (23)
*S. lugdunensis*	4 (4)
Streptococcus species	14 (13)
Enterococcus species	8 (8)
Corynebacterium species	5 (5)
Aerobic gram-negative bacteria	17 (16)
Enterobacter	7 (7)
Pseudomonas species	3 (3)
Anaerobic bacteria	7 (7)
Peptococcus species	2 (2)
Culture negative	5 (5)

The indications for PUR bearing surface exchange were the same as for DAIR. Contraindications to DAIR were: (1) presence of a sinus tract; (2) the prosthesis was loose; and/or (3) the wound could not be closed. A one-stage or 2-stage revision was performed for these indications. All patients meeting the inclusion criteria for aPUR DAIR were included, and no patient meeting the inclusion criteria was excluded. The McPherson classification for periprosthetic infection was used [26]. Forty-six (43%) infections were Type I, 7 (7%) were Type II, and 53 (50%) were Type III. The systemic host grade was A for 69 (65%) and B for 37 (35%). No patients were host grade C in this study. The lower extremity grade was 1 for 57 (54%) patients and 2 for 49 (46%) patients.

An aPUR bearing insert exchange was performed for total hip arthroplasty (n = 49), total knee arthroplasty (n = 50), hip resurfacing (n = 2), unicompartmental knee arthroplasty (n = 2), and hip hemiarthroplasty (n = 3). Both primary and revision cases were treated. In each instance, only the bearing surface was changed. For hip hemiarthroplasty, an aPUR head was used.

### Surgical procedure

**Figure 3:**
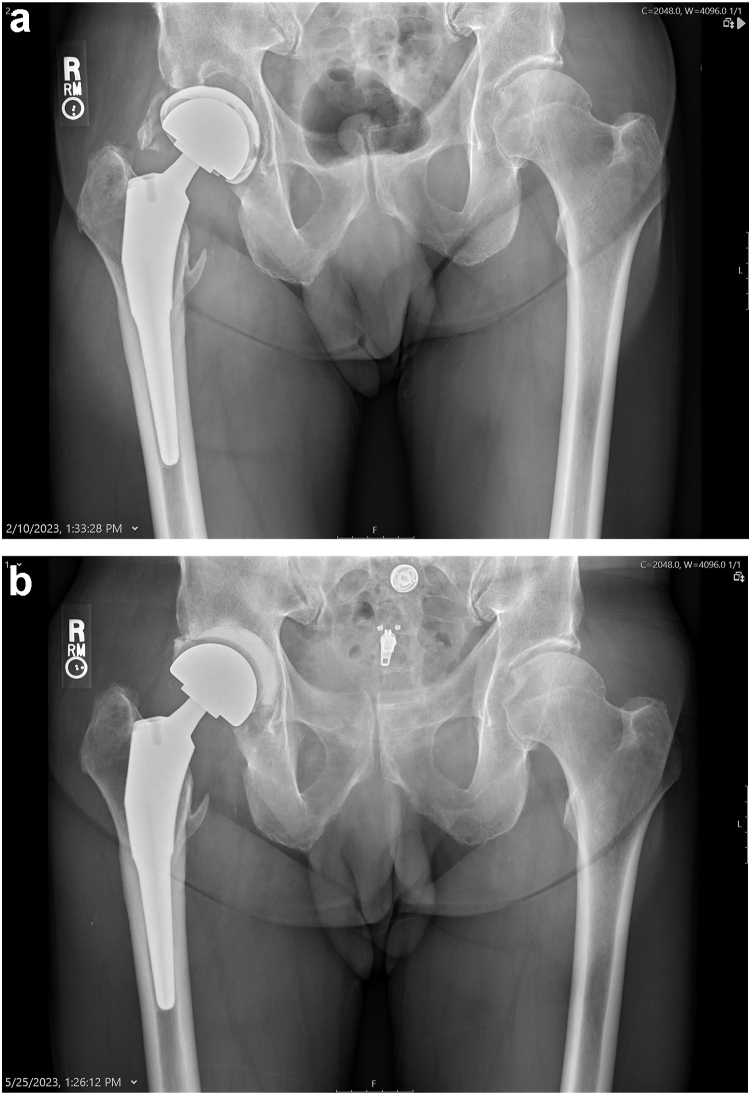
(a) Photograph of a chronic S. lugdunensis infection of a total hip arthroplasty in a 76-year-old man. (b) The postoperative anteroposterior pelvis radiograph shows the acetabular shell has been removed and replaced with an antibiotic-containing PUR liner supported by antibiotic cement.

The surgical procedure consisted of an arthrotomy and a complete synovectomy. The PE component was removed, and pulse lavage irrigation was performed using several liters of saline. The PE component was exchanged for an aPUR component. In 3 instances, the acetabular shell was also removed, and the revision PUR antibiotic liner was secured to the pelvis with antibiotic bone cement. This was done because of a lytic pocket of infection behind the shell (Fig. 3a and b). No femoral stems, knee tibial, or femoral metal components were removed. Culture-specific or vancomycin for culture-negative intravenous antibiotics were provided only before the implants were placed. No patients were placed on oral or intravenous antibiotics postoperatively. The joints were aspirated 180 days postoperatively, and cultures were held for growth for 10 days.

### Analysis of results

The Harris hip score, Knee Society Score, and patient acceptable symptomatic state (PASS) were used to assess functional outcomes [27]. The PASS question was: “Taking into account your joint pain and function and how it affects your daily life, including your ability to participate in sport and social activities, do you consider your current state acceptable?” All values were reported as means. Excel (Microsoft, Redmond, WA) and SAS v 9.4 (SAS Institute, Cary, NC) were used for data analyses.

## Results

### Clinical results

All patients treated achieved remission of their PJI, as determined by resolution of clinical symptoms, normalization of laboratory parameters, joint aspirations showing clear joint fluid, and negative cultures of aspirates. There were no signs of antibiotic toxicity or adverse drug reactions. No implants loosened, and none showed signs of wear. The mean Harris hip score was 93 (range, 88-99), the mean Knee Society Score was 90 (range 84-96), and 68% of the patients achieved PASS.

### Drug elution

Both serum and synovial fluid levels of antibiotics were measured (Table 2). The numbers for cement and intra-articular infusion were derived from patients evaluated for this study. There were 12 patients tested with complete antibiotic cement spacers treated without postoperative and intravenous antibiotics. There were also 12 patients treated with conventional DAIR procedures and PE exchange, followed by intravenous antibiotic therapy. The maximum systemic concentration was 0.15 μg/ml-0.26 μg/ml compared to the long-term systemic threshold for toxicity of 2 μg/ml as outlined by Goodman and Gillman [28]. The joint fluid bioavailability of vancomycin given intravenously was 20% of serum [28,29].

**Table 2:** Intra-articular and serum antibiotic levels μg/ml mean (SD).

Sample	Intra-articular		Serum	
	1 wk	3 wk	1 wk	3 wk
	Vanco/Tobra	Vanco/Tobra	Vanco/Tobra	Vanco/Tobra
aPUR	212 (75) / 168 (68)	168 (38) / 161 (39)	8 (2) / 4 (.5)	6 (.5) / 4 (.5)
aPE	171 (56) /123 (38)	122 (24) / 133 (42)	6 (1) / 4 (.5)	4 (.6) / 3 (1)
Antibiotic cement	322 (81) / 272 (61)	54 (13) /31 (12)	2 (.7) / 3 (.5)	.5 (.5) / .4 (.4)
Intra-articular infusion	2062 (172) / 1706 (232)	2078 (184) / 1806 (242)	2 (.5) / 2 (.5)	3 (1) / 4 (1)

Vanco, Vancomycin; Tobra, Tobramycin.

### Wear properties

Wear testing was also conducted for aPUR, antibiotic bone cement, and PE with or without antibiotics. After 10 million cycles, the mean wear rate for aPUR with vancomycin or tobramycin antibiotic added was 19 ± 9 mm^3^/million cycles and 25 ± 11 mm³/million cycles for a 46 mm acetabular component. The wear rate for a highly cross-linked PE tibial implant was from 13 ± 1.7 to 47 ± 9 mm^3^/million cycles (Table 3). The elongation at break, ultimate tensile strength, yield strength, and tensile toughness for aPUR are shown in Table 4.

**Table 3:** Gravimetric wear simulator testing results for tibial and acetabular bearings expressed as mean in mg/million and (SD).

Sample	Tibial insert	Acetabular insert
aPUR	23 (9)	46 (11)
aPE	33 (12)	29 (15)
Bone cement	880 (92)	900 (111)
Virgin PUR	12 (8)	15 (7)
Virgin PE	21 (7)	13 (6)

**Table 4:** Mechanical (tensile) properties: elongation at break, ultimate tensile strength, yield strength, and tensile toughness.

Sample	Mean EAB % (SD)	Mean UTS MPa (SD)	Mean YS MPa (SD)	Mean TT J/M^3^ (SD)
Antibiotic PUR	268 (15)	51 (3)	17 (1)	56 (6)
Antibiotic PE	380 (13)	28 (1)	18 (1)	68 (3)
Antibiotic cement	2 (.5)	47 (.5)	N/A	1
Virgin PUR	292 (17)	66 (6)	21 (3)	70 (10)
Virgin PE	397 (11)	56 (4)	23 (3)	88 (9)

EAB, elongation at break; UTS, ultimate tensile strength; YS, yield strength; TT, tensile toughness.

The average particle size was 0.50 μm for PUR compared to 0.52 μm for PE, but there were 77% fewer submicron particles for PUR compared to cross-linked PE [30]. The resistance to wear of the aPUR was substantially higher when compared to gentamicin-loaded bone cement. The wear rate of aPUR was approximately 20 times lower than that of bone cement as an articulation.

## Discussion

The overall goal of this study was to evaluate a simpler, less traumatic, and more patient-centric alternative to 1-stage or 2-stage explant and reimplantation surgery to treat PJI. Currently, it is questionable whether a 2-stage revision is the gold standard because of its known complications and challenges [2,5,11]. Also, staged reimplantation is not always effective. This goal was achieved by providing a more effective DAIR technique to treat PJI. There have been no failed implants in this study with a follow-up of 26 years.

This study found that aPUR acetabular and tibial inserts are safe and effective in the treatment of PJI. PUR has been used and evaluated extensively. It has strong mechanical properties, low wear, and excellent biocompatibility [19]. The strong wear and mechanical characteristics of PUR are not degraded by the addition of antibiotics. This study found that aPUR achieved and maintained remission of PJI and that all patients responded fully and without complications to the treatment. Also, the treatment was more convenient, efficient, and cost-effective.

PUR containing antibiotics works well and is a better alternative to a cement spacer or an antibiotic cemented all-PE tibial and acetabular prosthesis. The elution characteristics are favorable compared to cement antibiotic spacers. The release of antibiotics is high enough to penetrate biofilm. The release is maintained much longer compared to antibiotic cement, and the wear resistance is 20 times less than antibiotic cement. There were no local or systemic complications or toxicities; none would be expected, as the levels measured in the serum were below the toxic range. The wear characteristics of the acetabular and tibial inserts are consistent with a lifetime of use.

Antibiotics have been placed in bone cement since it was first used in 1953 [7]. Patients who were treated with PUR bone glue received embedded antibiotics when treating an open fracture [17]. Also, successful revision implant surgery for PJI was performed using aPUR starting in 1959 [14,20]. There were no known toxicities, but the success was not reported because the initial PURs were no longer used after 1962 [18]. The concerns about PUR were due to its inconsistent outcomes with fracture healing and spinal fusion [18]. The PUR preparations used as an articulating surface in the present study are different from the preparations used in the early applications.

Polyethylene has been the most widely used bearing surface for total joint arthroplasty. This is based on historical familiarity rather than the superiority of this polymer over other choices [31]. Polyethylene can also be used as a carrier for antibiotics [13,32]. This has been studied extensively in the laboratory and in animal models with successful results. At the present time, there is no information available about its use in patients, yet the expectation is that antibiotic PE will also be an effective strategy for PJI [3,4]. The wear resistance of aPUR was similar to that of the experimentally prepared PE containing antibiotics.

There are limitations to this work. One hundred six patients were treated in this study. As infection studies always are, this was a heterogeneous group. The severity of the infections varied, as did the health of the patients. Nevertheless, all patients achieved remission, and all patients had secure metal components. Infections are a balance between the biological burden of the infection and the immune system of the patient.

It is important to use local rather than systemic antibiotics. Intravenous antibiotics meet the minimum inhibitory concentration in tissue, but the key issue is the minimum biofilm eradication concentration rather than the tissue concentration. Antibiotic cement achieves very high levels but does not always persist long enough. Intra-articular infusions are also highly effective and provide levels high enough to penetrate biofilm, but they require an implanted catheter and frequent dosing [11].

Another limitation of this study is that all patients were treated by one surgeon. It is likely that a group larger than 106 patients will have cases that do not achieve and maintain remission. The functional outcomes are similar to primary joint arthroplasty outcomes. The PASS scores are high, with typical total hip PASS scores of 48 to 54; this may be in part due to the gratitude patients have for retaining their implants [1,5,10].

A future goal would be to provide bacterial- and wear-resistant, mechanically strong bearing surfaces for routine use. This study focused on providing patients with a less difficult and more effective treatment for PJI. This is a promising approach to a very difficult problem faced by an unfortunate few of our joint implant patients.

## Conclusions

Delivery of antibiotics by using drug eluting polyurethane tibial and aceteabular inserts is a novel, simple, and useful method of treating an infected joint replacement. Polyurethane as a bearing surface has excellent bicompatibility and wear characteristics. Safe and sustained bactericidal levels of antibiotics are released. This method improves patient's quality of life during treatment and has fewer complications than more commonly used 1 or 1-stage treatments using antibiotic bone cement and intravenous antibiotics.

## Conflicts of interest

The author declares there are no conflicts of interest.

For full disclosure statements refer to https://doi.org/10.1016/j.artd.2024.101409.

## CRediT authorship contribution statement

**James W. Pritchett:** Writing – original draft, Project administration, Methodology, Data curation, Conceptualization.

## Appendix A Supplementary Data

Conflict of Interest Statement for Pritchett

## References

[1] Bryan A.J., Abdel M.P., Sanders T.L., Fitzgerald S.F., Hanssen A.D., Berry D.J. (2017). Irrigation and debridement with component retention for acute infection after hip arthroplasty. J Bone Joint Surg Am.

[2] Patel R. (2023). Periprosthetic joint infection. N Engl J Med.

[3] Gil D., Atici A.E., Connolly R.L., Hugard S., Shuvaev S., Wannomae K.K. (2020). Addressing prosthetic joint infections via gentamicin-eluting UHMWPE spacer. Bone Joint J.

[4] Suhardi V.J., Bichara D.A., Kwok S., Freiberg A.A., Rubash H., Malchau H. (2017). A functional drug-eluting joint implant. Nat Biomed Eng.

[5] Moore A.J., Blom A.W., Whitehouse M.R., Gooberman-Hill R. (2015). Deep prosthetic joint infection: a qualitative study of the impact on patients and their experiences of revision surgery. BMJ Open.

[6] Mallon C., Gooberman-Hill R., Blom A., Whitehouse M., Moore A. (2018). Surgeons are deeply affected when patients are diagnosed with prosthetic joint infection. PLoS One.

[7] Haboush E.J. (1953). A new operation for arthroplasty of the hip based on biomechanics, photoelasticity, fast-setting dental acrylic, and other considerations. Bull Hosp Joint Dis.

[8] Choi H.R., Freiberg A.A., Malchau H., Rubash H.E., Kwon Y.M. (2014). The fate of unplanned retention of prosthetic articulating spacers for infected total hip and total knee arthroplasty. J Arthroplasty.

[9] Struelens B., Claes S., Bellemans J. (2013). Spacer-related problems in two-stage revision knee arthroplasty. Acta Orthop Belg.

[10] Pritchett J.W. (2020). Decision matrix-guided treatment of infected hip resurfacing. J Long Term Eff Med Implants.

[11] Whiteside L.A., Roy M.E., Nayfeh T.A. (2016). Intra-articular infusion: a direct approach to treatment of infected total knee arthroplasty. Bone Joint J.

[12] Pritchett J.W. (2016). Very large diameter polymer acetabular liners show promising wear simulator results. J Long Term Eff Med Implants.

[13] Wahlig H., Dingeldein E., Buchholz H.W., Buchholz M., Bachmann F. (1984). Pharmacokinetic study of gentamicin-loaded cement in total hip replacements. Comparative effects of varying dosage. J Bone Joint Surg Br.

[14] Petis S.M., Perry K.I., Pagnano M.W., Berry D.J., Hanssen A.D., Abdel M.P. (2017). Retained antibiotic spacers after total hip and knee arthroplasty resections: high complication rates. J Arthroplasty.

[15] Pritchett J.W. (2008). Curved-stem hip resurfacing: minimum 20-year followup. Clin Orthop Relat Res.

[16] Townley C.O., Frisch C.K., Sendijarevic A., Inventors; BioPro Inc., assignee (2001).

[17] Mandarino M.P., Salvatore J.E. (1959). Polyurethane polymer; its use in fractured and diseased bones. Am J Surg.

[18] Redler I. (1962). Polymer osteosynthesis. A clinical trial of ostamer in forty-two patients. J Bone Joint Surg Am.

[19] Elsner J.J., Shemesh M., Mezape Y., Levenshtein M., Hakshur K., Shterling A. (2011). Long-term evaluation of a compliant cushion form acetabular bearing for hip joint replacement: a 20 million cycles wear simulation. J Orthop Res.

[20] Pritchett J.W. (2020). Total articular knee replacement using polyurethane. J Knee Surg.

[21] ASTM International. ASTM F732-17 (2017). Standard test method for wear testing of polymeric materials used in total joint prostheses. http://www.astm.org.

[22] (2002). Implants for surgery-wear of total hip joint prostheses-Part 1: loading and testing machines and corresponding environmental conditions for the test.

[23] (2014). Implants for surgery-wear of total knee joint prostheses-Part 3: loading and displacement parameters for wear-testing machines with displacement control and corresponding environmental conditions for test.

[24] ASTM International. ASTM F2003 (2017) (2017). Standard practice for accelerated aging of Ultra-high molecular weight polyethylene after gamma irradiation in air. http://www.astm.org.

[25] ATSM International. ASTM F2025-06 (2018). Standard practice for gravimetric measurement of polymeric components for wear assessment. http://www.astm.org.

[26] McPherson E., Tontz W., Patzakis M., Woodsome C., Holtom P., Norris L. (1999). Outcome of infected total knee utilizing a staging system for prosthetic joint infection. Am J Orthop (Belle Mead NJ).

[27] Paulsen A., Roos E.M., Pedersen A.B., Overgaard S. (2014). Minimal clinically important improvement (MCII) and patient-acceptable symptom state (PASS) in total hip arthroplasty (THA) patients 1 year postoperatively. Acta Orthop.

[28] Goodman L., Gilman A., Brunton L., Chabner B., Knollmann B. (2011). The Pharmacological basis of therapeutics.

[29] Lloyd K.C., Stover S.M., Pascoe J.R., Baggot J.D., Kurpershoek C., Hietala S. (1988). Plasma and synovial fluid concentrations of gentamicin in horses after intra-articular administration of buffered and unbuffered gentamicin. Am J Vet Res.

[30] (2010). Wear of implant materials-polymer and metal wear particles – isolation and characterization.

[31] Scales J.T. (1966). Arthroplasty of the hip using foreign materials: a history. Proc Inst Mech Eng.

[32] Pritchett J.W., Bortel D.T. (1991). Tobramycin-impregnated cement in total hip replacements. Orthop Rev.

